# Resurrection of Dormant *Daphnia magna*: Protocol and Applications

**DOI:** 10.3791/56637

**Published:** 2018-01-19

**Authors:** Maria Cuenca Cambronero, Luisa Orsini

**Affiliations:** ^1^Environmental Genomics Group, School of Biosciences, University of Birmingham

**Keywords:** Environmental Sciences, Issue 131, Resurrection biology, waterflea, dormancy, longitudinal data, common garden experiments, competition experiments

## Abstract

Long-term studies enable the identification of eco-evolutionary processes that occur over extended time periods. In addition, they provide key empirical data that may be used in predictive modelling to forecast evolutionary responses of natural ecosystems to future environmental changes. However, excluding a few exceptional cases, long-term studies are scarce because of logistic difficulties associated with accessing temporal samples. Temporal dynamics are frequently studied in the laboratory or in controlled mesocosm experiments with exceptional studies that reconstruct the evolution of natural populations in the wild.

Here, a standard operating procedure (SOP) is provided to revive or resurrect dormant *Daphnia magna*, a widespread zooplankton keystone species in aquatic ecosystems, to dramatically advance the state-of-the-art longitudinal data collection in natural systems. The field of Resurrection Ecology was defined in 1999 by Kerfoot and co-workers, even though the first attempts at hatching diapausing zooplankton eggs date back to the late 1980s. Since Kerfoot's seminal paper, the methodology of resurrecting zooplankton species has been increasingly frequently applied, though propagated among laboratories only via direct knowledge transfer. Here, an SOP is described that provides a step-by-step protocol on the practice of resurrecting dormant *Daphnia magna* eggs.

Two key studies are provided in which the fitness response of resurrected *Daphnia magna* populations to warming is measured, capitalizing on the ability to study historical and modern populations in the same settings. Finally, the application of next generation sequencing technologies to revived or still dormant stages is discussed. These technologies provide unprecedented power in dissecting the processes and mechanisms of evolution if applied to populations that have experienced changes in selection pressure over time.

**Figure:**
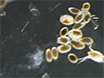


## Introduction

Long-term studies are critical to understanding ecological and evolutionary processes in nature and in assessing how species respond to and persist during environmental change^1^. This is because eco-evolutionary processes happen across generations and changes in the environment occur over long time spans. Furthermore, long-term studies provide key empirical data that improve the accuracy of predictive modeling to forecast evolutionary responses of natural ecosystems to environmental changes^2^. The accuracy of these models is critical to implement management and conservation strategies to preserve biodiversity and ecosystem services.

Excluding a few exceptional cases (*e.g.*, Galapagos Darwin finches^3^ and algae^4^), long-term studies are largely limited to species with short generation time that can be propagated in the laboratory^5^,^6^,^7^,^8^. Hence, the processes underpinning evolutionary dynamics remain elusive. Because of logistic difficulties associated with accessing temporal samples, empirical data are studied more frequently in a spatial than in a temporal context, and temporal eco-evolutionary processes are inferred or modeled from spatial data. This approach is known as 'space-for-time' substitution^9^, whereby space is adopted as a surrogate to study temporal evolutionary dynamics. The main limitation of the 'space-for-time' substitution is that rates of adaptation at different spatial scales differ from temporal variation in the same population; hence, inferences based on replacement of time with space are biased^10^.

A powerful alternative that allows studying evolutionary dynamics in natural ecosystems over time is the analysis of ecological and genetic changes in species producing dormant stages^11^. These dormant stages accumulate to form stratified biological archives that can be accurately dated and paleolimnologically characterized^12^,^13^. Importantly, these dormant stages can be resuscitated and used in laboratory experiments, where their evolutionary response to environmental change can be directly measured. Historical populations can be competed against their modern evolved descendants to study fitness changes and the function of genes evolving in step with environmental change^14^,^15^,^16^.

Dormant stages include seeds, cysts, spores, and egg banks. Although the first studies on resuscitated dormant eggs dates back to the late 1980s^17^, and a handful of studies have applied this technique in the early 1990s^18^,^19^, the field of Resurrection Ecology has been formally established by the seminal paper of Kerfoot and co-workers in 1999^20^. This practice has been applied mainly in paleolimnological reconstructions of freshwater species^17^,^21^,^22^. However, a SOP is not yet available. Here, a step-by-step description of the resurrection protocol applied to dormant eggs of the zooplankton species *Daphnia magna* is provided, from the sampling of sediment to the establishment of clonal cultures from hatchlings. Steps of the SOP that are readily transferable to other species of *Daphnia*, as well as steps that may require additional optimization, are discussed.

*Daphnia* are freshwater zooplankters present in the majority of lotic habitats^23^ . *Daphnia* species are either obligate asexual or cyclical parthenogens. *D. magna* is a cyclical parthenogen that reproduces clonally under favorable environmental conditions^24^. When environmental conditions deteriorate, male production occurs and sexual recombination leads to the formation of fertilized eggs that enter a state of dormancy protected from the environment by a chitin case called ephippium. A proportion of these dormant eggs hatch when favorable environmental conditions return. However, a large proportion of the dormant egg bank never has a chance to hatch and thus build up biological archives over time. Dormant stages remain buried in the sediment of lakes and ponds and can be resurrected for the study of evolutionary dynamics over extended time periods. Because dormant eggs of *D. magna* are the result of sexual recombination, they are a good representation of the natural genetic diversity of the species^25^. Moreover, they can be maintained via clonal reproduction in the laboratory. These characteristics provide the unique advantage of isogenic model organisms, while retaining the natural genetic diversity.

Two key studies are presented to demonstrate the advantages of directly comparing historical and modern descendants of the same population of *D. magna* experiencing environmental selection pressure over time. *D. magna* specimens were resurrected from Lake Ring (Denmark), a shallow (5 m depth; surface 22 ha) mixed pond that has experienced an increase in average temperature and heat waves occurrence over time. *D. magna* (sub)populations were resurrected along this temporal gradient spanning 60 years (1960–2005) and studied to investigate evolutionary response to temperature warming. In the first study in a common garden experiment, changes in fitness-linked life history traits were measured in response to an increase in temperature of +6 °C, in line with the predictions of the Intergovernmental Panel for Climate Change for the upcoming 100 years^26^. In the second study, a mesocosm experiment was used to measure the competitive abilities of the three (sub)populations under warming. These experiments combined show that in presence of warming as the only stress, all life history traits and populations show a high level of plasticity and have equal competitive abilities. These findings suggest that warming as a single stress does not impose significant fitness costs, at least in the population studied here.

## Protocol

The following SOP provides a step-by-step description of the protocol used to resurrect *Daphnia magna* dormant eggs, including a detailed description of sampling, isolation of ephippia from the sediment, and establishment of clonal cultures (Figure 1).

**Figure:**
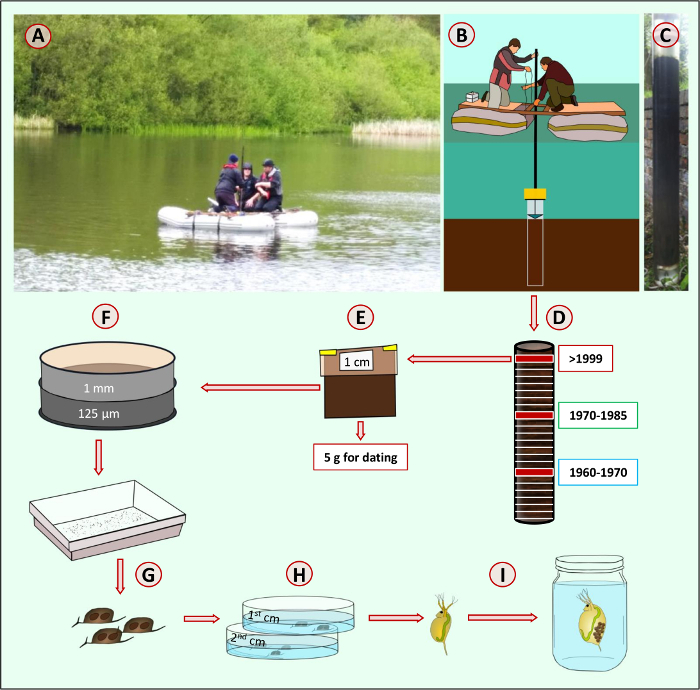

**Figure 1: Step-by-step guide to resurrection of *Daphnia magna. ***Sediment from a natural freshwater habitat (**A**) is sampled with a piston corer (**B**). The sediment core (**C**) is sliced in incremental layers of 1 or 0.5 cm (**D**). Each layer of sediment is stored in a sample zip lock bag (**E**) in dark and cold conditions (4 °C). Each layer of sediment is weighed and sieved using geological sieves (1 mm and 125 µm mesh sizes, **F**). White background trays are used to isolate *Daphnia magna* ephippia (**G**). Decapsulated dormant eggs (**H**) are transferred to Petri dishes and exposed to light and temperature stimuli to induce hatching. Hatchlings are transferred to separate jars (**I**) to establish isoclonal lines. Please click here to view a larger version of this figure.

### 1. Sampling of Sediment Cores

1. Sample sediment from lakes or ponds using a piston corer. This protocol used Big Ben^27^, a core tube of approximately 1.5 m in length with an internal tube diameter of 14 cm. Big Ben consists of a piston on a rope and a corer head, to which rods are attached to drive the tube into the sediment. A core catcher aids the support of the core tube when full of sediment. To extrude the sediment, a framework keeps the core tube upright and stationary, and a modified bottle jack is used to push the piston upwards during the extrusion process (**Supplementary Video 1**).
  1. For shallow ponds of less than 1 m in depth, use a plexiglass gravity corer of no more than 6 cm of diameter manually pushed into the sediment.
  2. For deep lakes (>6 m of depth), use Livingston piston corers^28^ or single-drive Griffith sediment corers with the aids of an anchored pontoon boat. The Livingstone-type drive rod piston corer can be used in water up to about 30 m deep to collect successive one-meter drives of soft to consolidated lake sediment. The single-drive Griffith corer consists of a simple but robust core head that connects standard polycarbonate tubes to Livingstone drive rods. The corers are pushed into the sediment with the rods, and a piston provides the suction needed for the recovery of sediment (Figure 2).
  3. For retrieving continuous, undisturbed cores, use vibracoring. These corers work on a variety of water depths and can retrieve core samples of different lengths, depending on sediment lithology. Low amplitude vibration that is transferred from the vibracore head down through the attached barrel or core tube liquefies sediments, enabling the core barrel attached to the vibracore unit to penetrate into the liquefied sediments. Some vibracorers are small, lightweight, and portable, others are large heavy units that can only be deployed from large vessels. The choice of corers depends on the lithology of sediment.
2. Slice the core horizontally in incremental layers of 1 cm or less using a flat metal surface (**Supplementary Video 1**). Sediment corers like the one used here are designed to reduce hydrostatic pressures at extrusion, reducing disturbance of the sediment layers. When other corers are used, the outer rind of each sediment layer may be removed with a cookie-cutter sort of blade to limit contamination among layers.
3. Collect each sediment layer in a separate sampling bag (**Supplementary Video 1**), and store in dark and cold (4 °C) conditions.
4. Collect a minimum of 5 g of sediment from all layers for radiometric dating. As radiometric dating is an established protocol for a detailed description of the dating assay, refer to existing publications^12^,^13^.

**Figure:**
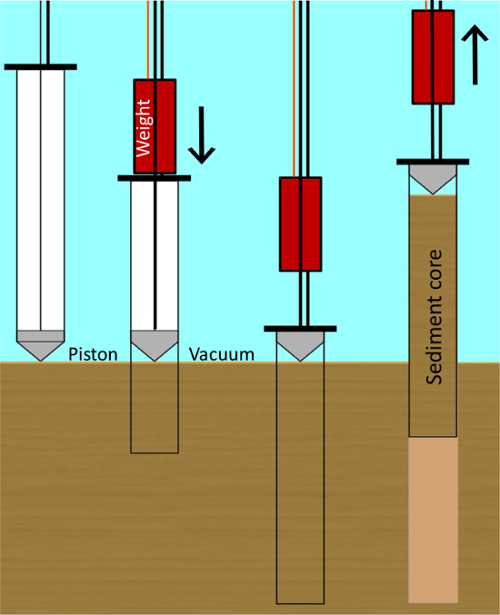

**Figure 2: Cartoon of the piston coring procedure. **Piston corer, a hollow tube with an internal sliding seal (the piston) that produces a weak vacuum. When the piston touches the sediment-water interface, the weight pushes the core barrel into the sediment and the vacuum causes the sediment being cored to enter and move up the tube without disturbing the sediment layers. Please click here to view a larger version of this figure.

### 2. Sieving of Sediment Layers

1. Using a precision scale, weigh each sediment layer for future reference. Use the surface area and weight to calculate the species density in the lake.
2. Sieve each sediment layer using two geological sieves piled on top of each other. The first sieve has a mesh size of 1 mm and separates clay, large invertebrates and particulate matter, *e.g.* seeds, plants, and insects, from the remainder of the sediment. The second sieve has a mesh of 125 µm and separates *D. manga* ephippia and small particulate from the remainder of the sediment (**Supplementary Video 2**).
3. **Transfer small aliquots of the sediment fraction collected onto the 125 µm mesh sieve to a white background tray. Depending on the type of sediment, smaller or larger aliquots of sediment may be transferred at each time.**
  1. Add small volumes (up to 200 mL) of medium to the sampling white tray to re-suspend the transferred sediment fraction and facilitate eye spotting of ephippia (Figure 3). The medium used to resuspend the sediment can be dechlorinated tap water, borehole water, COMBO^29^, or ADaM medium (Aachener Daphnien)^30^. Hereafter, the term 'medium' will be used to refer to either or all the listed solutions.

**Figure:**
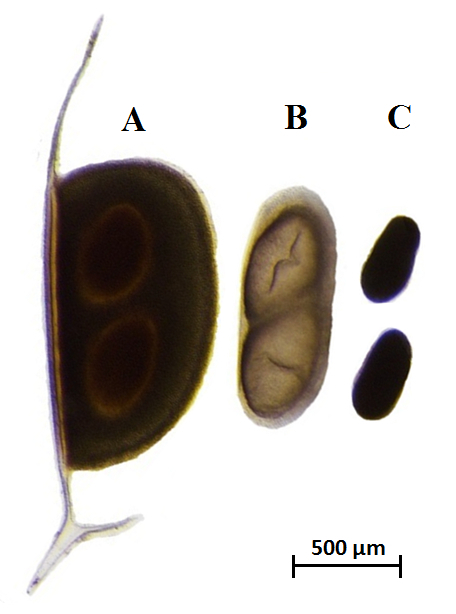

**Figure 3: *Daphnia magna* ephippium. **Dormant *Daphnia magna* eggs immediately after decapsulation. The ephippium (**A**), the inner egg membrane (**B**), and the dormant eggs (**C**) are shown*. *Scale bar = 500 µm.

### 3. Decapsulation of Ephippia and Hatching

1. With a disposable Pasteur pipette or using microdissection forceps, transfer individual ephippia to Petri dishes filled with 10 mL of medium. Use at least a Petri dish per layer of sediment.
2. Under a stereo microscope, decapsulate each ephippia using microdissection forceps by forcing open the chitin case (**Supplementary Video 3**). Remove the resting egg inner membrane delicately, paying attention to not disrupt the eggs, and transfer them to the Petri dish filled with medium using a Pasteur pipette. Decapsulation increases hatching success in *D. magna;* however, it may not be required or it may be challenging for other *Daphnia* species producing smaller ephippia.
3. Expose the decapsulated eggs to a full spectrum long day photoperiod light (16:8 light:dark) and high temperature (20 ± 1 °C) to induce hatching in a controlled temperature device (incubator) or room. Hatching occurs between 48 h and several weeks (up to four; **Supplementary Video 4**). In absence of decapsulation, directly expose the ephippia to hatching stimuli (long day photoperiod light (16:8 light:dark) and high temperature (20 ± 1 °C)).

### 4. Establishing Isoclonal Lines of *Daphnia magna*

1. Establish isoclonal lines from single hatchlings by transferring individual *D. magna* from step 3.3 to separate jars filled with 200 mL of medium using a disposable Pasteur pipette. Each individual is genetically distinct, being the result of sexual recombination.
2. Maintain isoclonal lines indefinitely in stock conditions consisting of 10 ± 1 °C, 16:8 light:dark regime, fed weekly with 0.2 mg C/L of *Chlorella vulgaris* or other green algae (*e.g.*, *Scenedesmus obliquus*). Renew the medium every third week. Stock conditions may change with temperature, feeding regimes, and species.

### 5. Key Studies

NOTE: A description of two key studies is provided in which resurrected *D.magna* (sub)populations from the sedimentary archive of Lake Ring (Denmark) are used to assess the evolutionary response to warming. Three (sub)populations were resurrected from the following time periods: 1960-1970, 1970-1985, and >1999. *D. magna* hatching success from the sedimentary archive ranged between 11 and 58% (Figure 4). From the hatchlings obtained from each timeperiod, a random subset was chosen for the two key studies described here. These studies were designed to assess whether (sub)populations resurrected from different time periods along the temperature gradient showed differences in fitness-linked life history traits (5.1), and whether they had different competitive abilities (5.2) after exposure to warming.

**Figure:**
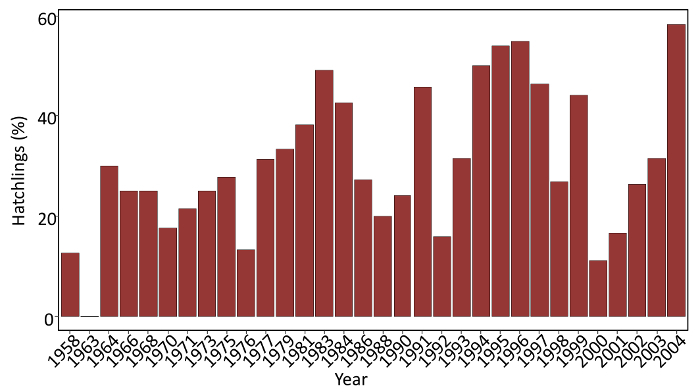

**Figure 4: Hatching success in a sedimentary archive sampled from Lake Ring. **The proportion of successful hatchlings along the sedimentary archive of Lake Ring used in the key studies. Please click here to view a larger version of this figure.

1. **Common garden experiments**
  1. Transfer ten hatchlings per (sub)population from stock cultures to common garden conditions: 16 °C; long photoperiod (16:8 light:dark regime); feed daily with 0.8 mg C/L of *Chlorella vulgaris*, and renew medium every second day. Maintain the hatchlings in common garden conditions for at least two generations (*ca.* 45 days). Common garden conditions reduce interference from the maternal effect and synchronize reproduction among hatchlings.
  2. Upon release of the second brood into the brood chamber, transfer adult females from the second generation to 500 mL jars filled with medium until they release the second brood of juveniles.
  3. Randomly transfer individual juveniles of 24–48 h, born from the second brood of the second generation, to 100 mL jars filled with medium and exposed to the following experimental conditions: 18 °C (current temperature in the lake) and 24 °C (warming, temperature forecasted by the IPCC 26 for the upcoming 100 years), 16:8 light:dark regime, and feed daily 0.8 mg C/L of *Chlorellavulgaris*.
  4. On each experimental animal, measure the size at maturity with a stereomicroscope as the distance between the head and the base of the tail spine (Figure 5). Photograph each animal and subsequently analyze its size using a suitable image software.
  5. Measure the age at maturity: the day in which eggs are observed for the first time in the brood chamber.
  6. Measure the mortality: the number of extinct individuals during the experiment.
  7. Measure the fecundity: the total number of offspring released in first and second clonal reproduction.
  8. Measure the population growth rate, estimated using the Euler's equation (1): 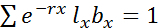 (1) Where *l_x_* is the proportion of survival at age *x*, *b_x_* is the number of neonates produced per surviving individual at age *x*, and r is the intrinsic rate of natural increase.
  9. Perform statistical analysis using commercially available software. Here, use R^31^ to plot reaction norms (phenotypic expression of a single genotype across environments) for each life history trait, using the ggplot2 package. Calculate mortality rates per population via survival analysis (R package rms; https://cran.r-project.org/web/packages/rms/rms.pdf). Finally, perform an analysis of variance (ANOVA, **Table 2**) in R^31^ to assess whether the effect of temperature on traits can be explained by evolution (differences among populations), plasticity (response to treatment), or evolution of plasticity (population x treatment).
2. **Competition experiment**
  1. Transfer ten hatchlings per population from stock cultures to common garden conditions: 20 °C, long photoperiod (16:8 light:dark regime), feed daily with 0.8 mg C/L of *Chlorella vulgaris, *and renew medium every second day. Maintain common garden conditions for at least two generations (*ca.* 45 days) to reduce interference from maternal effects.
  2. Randomly assign five juveniles of 24 to 48 h from the second brood of the second generations of each hatchling to experimental mesocosms (20 L plastic aquaria filled with medium), in triplicates, at a density of 10 animals/L.
  3. Expose the mesocosms to 24 °C, 16:8 L:D regime, and feed daily with 0.8 mg C/L of *Chlorellavulgaris* in a controlled temperature chamber or incubator for a minimum of four weeks (>3 clonal generations).
  4. Cull each mesocosm weekly, refreshing 10% of the medium to simulate a population dynamic that *Daphnia* may encounter in the natural environment. Impose culling by removing a known volume of medium and animals from each aquarium (1.2 L in this case), and by replacing the culled volume with fresh medium. Start the culling regime after the experimental animals reach maturity (day 10).
  5. At the end of the fourth week, sample 32 animals from each mesocosm to assess shifts in genotypic composition as compared to the initial inoculum.
    1. Place individual *Daphnia* in microcentrifuge tubes and remove excess liquid with the help of a Pasteur pipette.
    2. Flash freeze individual tubes in liquid nitrogen and store at -80 °C.
    3. Extract genomic DNA from single individuals using available protocols and following the manufacturer's instructions.
    4. Amplify the extracted DNA using a number of genetic markers sufficient to provide a unique multilocus genotype per hatchling. Here, 8 microsatellites arranged in a single multiplex (M01, **Table 1**) were used following established protocols^32^,^33^.
    5. Genotype amplified fragments on a fragment analyzer.
    6. Conduct fragment analysis with a commercial or freely available software using a suitable size standard.
    7. Assess genotypic composition at the end of the experiment by genotyping the 32 individuals with a set of microsatellite loci as described^33^, and calculating the frequency of each genotype at the end of the experiment as compared to the initial inoculum.

**Figure:**
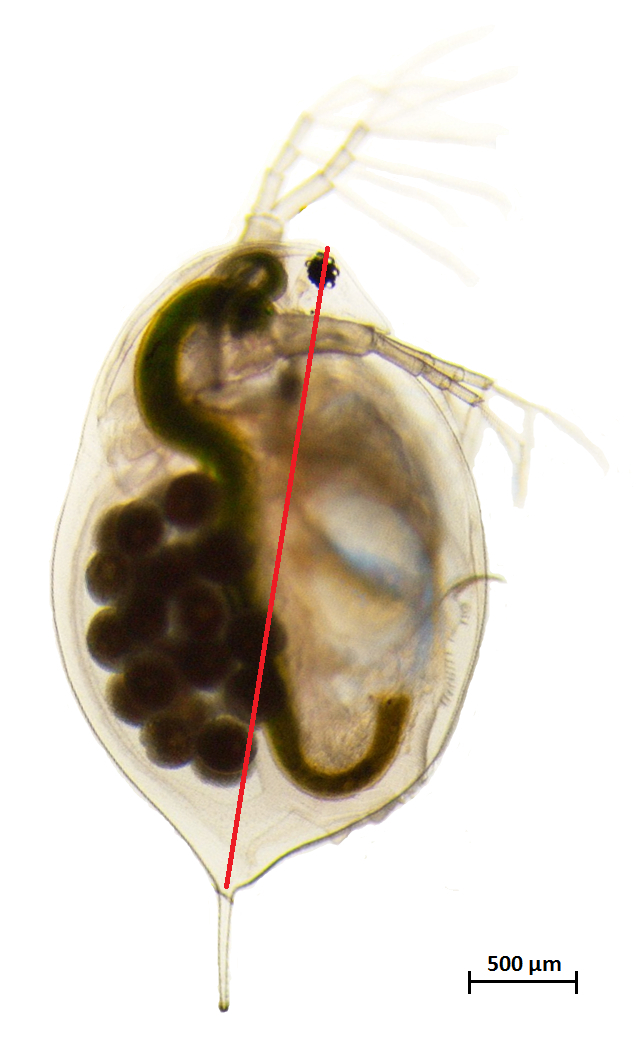

**Figure 5: Adult female *Daphnia magna. ***Adult female *Daphnia magna* with parthenogenetic eggs in the brood chamber. The distance between the head and the base of the tail spine is used to measure size of the animal. The red lines indicate the size measurements. Scale bar = 500 µm.

## Representative Results

Long-term empirical data are critical to the understanding of evolutionary dynamics and persistence of natural populations. Such data are generally challenging to obtain because of logistic difficulties associated with accessing temporal samples and the requirement of committing long-term to data collection. In the two key studies presented here, empirical evidence of the response to temperature of a central zooplankter in freshwater ecosystems is provided over evolutionary times. This is enabled by the use of layered dormant egg banks that provide the opportunity to study the response of historical populations and their modern descendants to environmental stress in common experimental settings.

**Common garden experiment** The common garden experiment showed that all life history traits responded to temperature (Figure 6 and****Figure 7). The ANOVA analysis revealed that all (sub)populations respond to temperature via plasticity (**Table 2**), except for mortality, which is unresponsive. Evidence of evolutionary changes (differences among (sub)populations) was observed only in population growth rate (**Table 2**), which significantly increased in two of the three (sub)populations at 24 °C (Figure 6).

**Figure:**
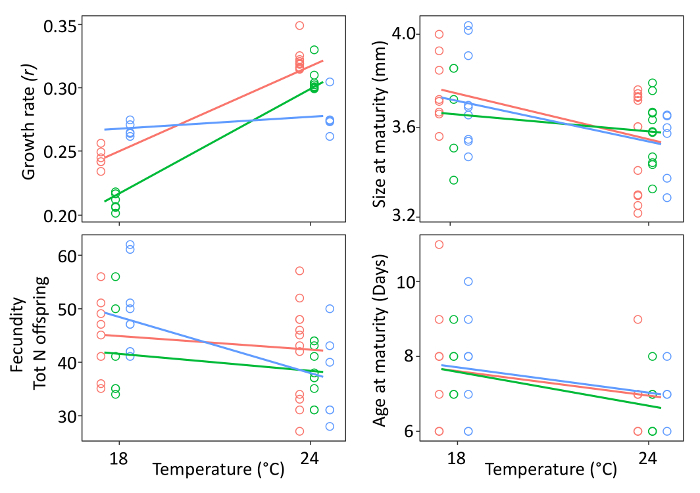

**Figure 6: Common garden experiment. **Reaction norms for life history traits (fecundity, size, and age at maturity) and population growth rate (*r*) are shown for each (sub)population under temperature warming (24 °C) as compared to the common garden and current temperature regime (18 °C). The population growth rate '*r*' is calculated using the Eulerian equation (1). Confidence intervals are shown. (Sub)populations are color coded: (i) blue: 1960-1970; (ii) green: 1970–1985; (iii) red: >1999. Please click here to view a larger version of this figure.

**Figure:**
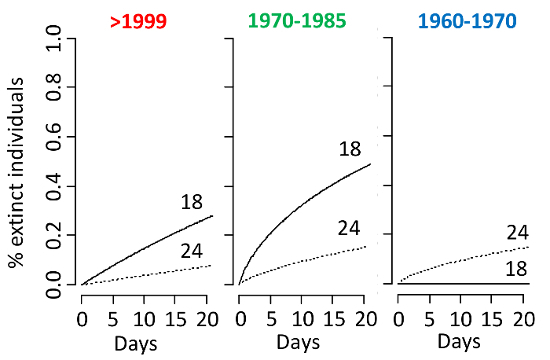

**Figure 7: Mortality. **Mortality rates per (sub)population (1960-1970; 1970-1985; >1999) are shown under warming (24 °C) as compared to modern temperature regimes (18 °C). Please click here to view a larger version of this figure.

**Mesocosm experiment** After four weeks of selection, represented by warming at 24 °C, the frequency of the three (sub)populations did not change significantly (χ^2 ^= 0.55, P = 0.76) as compared to the initial inoculum (Figure 8). Among the 30 genotypes inoculated in the mesocosm experiment, the majority was identified after four weeks of selection (Figure 9). Specifically, 70% of the inoculated genotypes were recovered, compatible with Poissonian expectation of recovering at least one representative of each genotype in a sample of 32 individuals.

**Figure:**
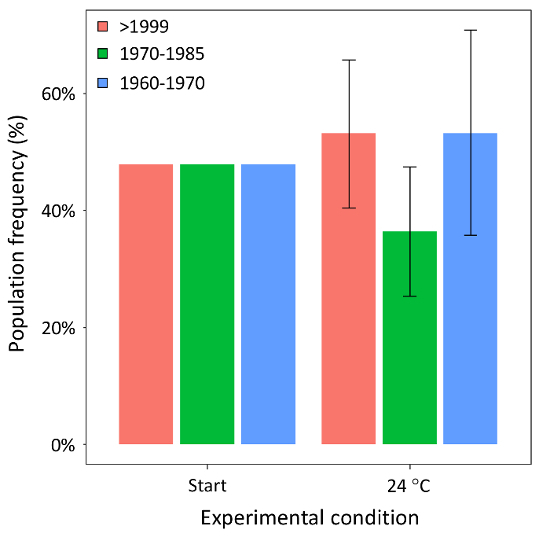

**Figure 8: Competition experiment - population frequency. **Population-averaged median and quartiles (25^th^ and 75^th^), is shown for the three (sub)populations of *D. magna* after four weeks of selection in mesocosm competition experiments (24 °C), as compared to an initial equal frequency (at the start). (Sub)populations are color coded as shown in Figure 6. Please click here to view a larger version of this figure.

**Figure:**
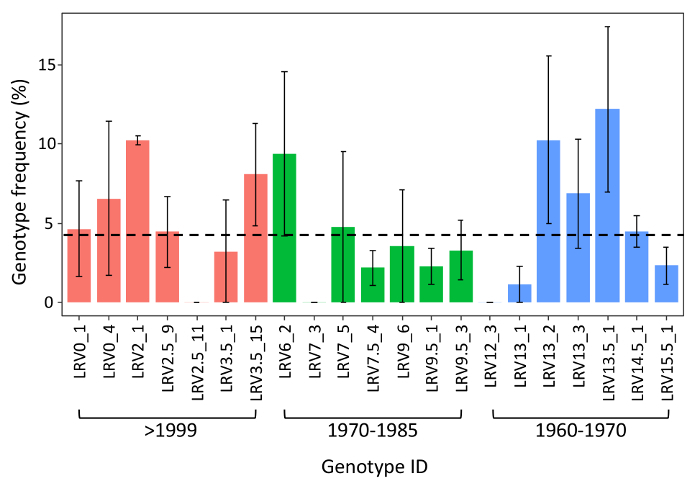

**Figure 9: Competition experiment - genotype frequency. **Genotype frequencies — averaged median and quartiles (25^th^ and 75^th^), are shown after four weeks of exposure to warming (24 °C) as compared to an initial equal frequency of genotypes (dotted line). Names on the x-axis are the inoculated genotypes ID, grouped per (sub)population (blue, 1960-1970; green, 1970-1985; red, >1999). Please click here to view a larger version of this figure.

**Table:** 

**Locus**	**AN**	**Size range (bp)**	**Primers (5’-3’)**	**Dye label**	**Repeat motif**	**Tm**
B008	HQ234154	150–170	F: TGGGATCACAACGTTACACAA	VIC	(TC)^9^	56
R: GCTGCTCGAGTCCTGAAATC
B030	HQ234160	154–172	F: CCAGCACACAAAGACGAA	PET	(GA)^11^	56
R: ACCATTTCTCTCCCCCAACT
B045	HQ234168	118–126	F: GCTCATCATCCCTCTGCTTC	NED	(TG)^8^	56
R: ATAGTTTCAGCAACGCGTCA
B050	HQ234170	234–248	F: TTTCAAAAATCGCTCCCATC	6FAM	(GAA)^6^	56
R: TATGGCGTGGAATGTTTCAG
B064	HQ234172	135–151	F: CTCCTTAGCAACCGAATCCA	6FAM	(TC)^8^	56
R: CAAACGCGTTCGATTAAAGA
B074	HQ234174	196–204	F: TCTTTCAGCGCACAATGAAT	NED	(GT)^9^	56
R: TGTGTTCCTTGTCAACTGTCG
B096	HQ234181	234–240	F: GGATCTGGCAGGAAGTGGTA	VIC	(AC)^15^	56
R: TTGAACCACGTCGAGGATTT
B107	HQ234184	250–274	F: GGGGTGAAGCATCAAAGAAA	PET	(CT)^8^	56
R: TGTGACCAGGATAAGAGAAGAGG

**Table 1: Microsatellite multiplex. **The NCBI Accession Number (AN), the multiplex information, the PCR primer sequences, the PCR size range, the repeat motif, the dye used to label the forward primer, and the annealing temperature (Tm) are shown.

**Table:** 

**Pop Growth rate (*r*)**	Df	F	P
Evolution (Pop)	2	30.309	***<0.001***
Plasticity (Temp)	1	531.546	***<0.001***
Evol. Plasticity (Pop x Temp)	2	65.137	***<0.001***
**Mortality**	Df	F	P
Evolution (Pop)	2	2.234	0.1162
Plasticity (Temp)	1	2.679	0.1071
Evol. Plasticity (Pop x Temp)	2	1.8657	0.164
**Fecundity **	Df	F	P
Evolution (Pop)	2	1.8852	0.1633
Plasticity (Temp)	1	6.8934	***0.0117***
Evol. Plasticity (Pop x Temp)	2	1.6511	0.203
**Size at maturity**	Df	F	P
Evolution (Pop)	2	0.211	0.8106
Plasticity (Temp)	1	11.1361	***0.0017***
Evol. Plasticity (Pop x Temp)	2	0.6586	0.5225
**Age at maturity**	Df	F	P
Evolution (Pop)	2	0.7811	0.4637
Plasticity (Temp)	1	8.0764	***0.0066***
Evol. Plasticity (Pop x Temp)	2	0.088	0.9159

**Table 2: Analysis of variance(ANOVA). **Analysis of variance testing whether changes in life history traits and population growth rate of the resurrected (sub)populations exposed to warming are explained by evolutionary adaptation (populations), plasticity (temperature treatment), and their interaction term (evolution of plasticity). Significant *p*-values (*p*<0.05) are shown in bold.

**Supplementary Video 1: Sampling of sediment cores. **The use of a Big Ben corer is shown. Big Ben is a core tube of approximately 1.5 m in length with an internal tube diameter of 14 cm. It consists of a piston on a rope and a corer head, to which rods are attached to drive the tube into the sediment. A core catcher is used to support the core tube that is deployed from a small vessel. The piston is pushed down into the sediment by gravitational pressure. A framework is used to support the core tube during the extrusion process carried out using a modified bottle jack that pushes the piston upwards. Each sediment layer is collected on a flat metal surface and transferred to transparent sampling bags for long term storage [dark and cold (4 °C) conditions]. Please click here to download this file.

**Supplementary Video 2: Sediment sieving. **The equipment required for sieving sediment is a precision scale, white sampling trays and geological sieves. From each sediment layer, at least 5 g are retained for radiometric dating. The remainder of the sediment is used to isolate ephippia. The sediment is sieved through two geological sieves, one with 1 mm and a second with 125 µm mesh size, piled on top of each other. Medium is poured on the 1 mm mesh sieve to separate clay, large invertebrates, and particulate matter. Medium poured on the second sieve with 125 µm mesh separates *D. magna* ephippia and small particulate matter. Aliquots of sediment are then transferred to a white sampling tray. *D. magna* ephippia are spotted by eye in the white background tray. Ephippia from each layer are collected in separate Petri dishes. Please click here to download this file.

**Supplementary Video 3: Decapsulation. **Under a stereomicroscope, *D. magna* ephippia are opened with microdissection forceps by applying pressure on the spine of the chitin case. The inner egg membrane is delicately removed and resting eggs gently transferred with a Pasteur pipette to a Petri dish containing 10 mL of medium. Please click here to download this file.

**Supplementary Video 4: Hatching. **After exposure to a long photoperiod and 20 °C, embryo development resumes between 48 h and few weeks. When development is complete, the embryos break free from the egg shell and freely swim in the medium. Please click here to download this file.

## Discussion

Due to the high thermal conductivity of water, freshwater ecosystems are at higher risk of biodiversity loss than terrestrial ecosystems in the face of global warming^34^. It is, therefore, critical to understand the response of keystone species in these ecosystems and identify coping mechanisms to survive thermal stress. The understanding of these mechanisms at the species and community levels can help predict how species are impacted by global warming and how the effect on individual species cascades to other trophic levels. Ultimately, understanding mechanisms of responses to global warming enables the identification of remediation strategies to mitigate extinctions.

The case studies presented here show that the response of *D. magna* to temperature increase is pervasively mediated by plasticity in life history traits and that response to temperature increase alone does not impose clear fitness costs, at least in the population studied here. High plasticity in life history traits is supported by non-significant differences in competing abilities of the (sub)populations in the presence of warming. However, longer-term competition experiments on multiple populations may be necessary to generalize these findings.

Resurrection of dormant stages provides an unprecedented resource to study mechanisms of adaptation and trajectories of a species' evolution through time^10^. Zooplankton species benefit from a rapid generation time (about 2 weeks), and the viability of dormant stages, which allows an ancestor to compete headtohead against its own descendants, or to 'replay' evolution that starts from various past states. Resurrection ecology essentially enables the investigation of whether a particular evolutionary outcome is contingent on some prior event. The identification of the genetic elements of evolution is currently possible in laboratory experiments using microorganisms for which 'ancestral lines' are frozen and resuscitated for comparative analysis with their evolved descendant^6^. However, one of the main limitations of working with laboratory organisms is that the 'ancestral state' is an already shifted baseline. The study of dormant stages allows the sampling of specimens from time predating any stress event (*e.g.*, pristine environmental conditions) and to measure evolutionary trajectories from undisturbed environmental conditions to various past states until modern times. In recent years, the study of DNA polymorphism in resurrected or still dormant zooplankton stages has provided important insights into past demographic and adaptive processes that have contributed to the genetic makeup of present-day populations^14^,^16^,^25^,^33^,^35^,^36^. With the higher accessibility of high throughput sequencing technologies, the genome and transcriptome of resurrected or still dormant stages can be sequenced and the type and number of genetic changes accumulated in evolving populations over time measured.

The resurrection SOP presented here has important applications in the field of multi-omics on two levels. Multi-omics technologies can be applied to resurrected specimens, allowing an exhaustive analysis of the molecular elements involved in adaptive responses to environmental selection pressure. In addition, omics technologies can be applied to decapsulated but still dormant stages. So far, the application of high throughput sequencing technologies to resting stages has been limited by the requirement of a large amount of input material. These limitations are being lifted^37^. With the lowering requirements for input material and progress in nanofluidics, whole genome sequencing (WGS) is now possible from as little as 1 ng or a few pg of starting material^38^. The use of whole genome amplification (WGA) and whole transcriptome amplification (WTA) techniques, enabling the enrichment of DNA and RNA from very small amounts of tissue, has revolutionized both metagenomics^39^,^40^ and medical research^41^. These technologies applied to decapsulated dormant eggs enable the exceeding of limitations associated with viability of dormant stages and the investigating of extended time periods (*e.g.*, centuries).

The resurrection of invertebrate communities producing resting stages enables the alignment of community histories with known changes in the natural landscapes, or with environmental changes inferred from analyses of the sediments orsoils^2^. The analysis of community changes in response to environmental change provides us with the ability to quantify eco-evolutionary feedbacks^42^ that have substantial consequences on population persistence^43^, trophic interactions^44^, community assembly^45^, and changes in ecosystem functions and services^46^. Finally, accurate predictions about biological responses to environmental change are paramount to guide the protection of biodiversity^47^. Current predictive models are inaccurate in this respect because they do not take into account important biological mechanisms such as demography, dispersal, evolution, and species interactions. Understanding how these processes change over time and using this information as a prior in forecast modelling will improve our ability to predict species and community persistence in the face of environmental change^2^.

The application of the SOP presented here is not without challenges. The primary limitation of resurrecting dormant stages is the need of specialized equipment for sampling. Additionally, the entire process, from sediment sieving to establishment of clonal cultures, requires considerable hands-on time.

Some of the SOP steps presented here are readily transferable to other *Daphnia* species. These are: sampling, establishment of clonal lines, and experimental design. However, other steps of the SOP may require further optimization tailored to the species under study. Decapsulation is often applied to *D. magna* specimens to improve hatching success. However, this approach may not be suitable for smaller specimens. Hatching stimuli can also vary among species^48^ and among conspecific specimen^49^. Hence, an *ad hoc* optimization of the hatching steps of the SOP may be required prior to applications to other crustaceans. Whilst the hatching success of the *D. magna* population resurrected from Lake Ring (30.5% across the sedimentary archive) is in line with previous results^49^, hatching success varies with the preservation state of the sediment, the species^50^,^51^, and the geographic origin of the sediment^48^. Future studies on the mechanisms that regulate entry and progression through the phases of diapause is required to identify optimal hatching stimuli tailored to different species.

Finally, background knowledge of the study system, in particular the presence of the species of interest over time, is advisable. This can be achieved via historical records. If historical records are not available, sampling and screening of the surface layers of the lake sediment prior to core sampling is advisable, although it may provide information only on the most recent history.

## Disclosures

The authors have nothing to disclose
